# The Parkinson’s Universe: A person-centered care model for Parkinson's disease

**DOI:** 10.1177/1877718X251399958

**Published:** 2025-12-03

**Authors:** Michael S Okun, Bastiaan R Bloem, E Ray Dorsey

**Affiliations:** 1 Norman Fixel Institute for Neurological Diseases, Department of Neurology, University of Florida Health, Gainesville, FL, USA; 2 Radboud University Medical Center, Department of Neurology, Donders Institute for Brain, Cognition and Behaviour, Nijmegen, the Netherlands; 3 Center for the Brain and the Environment, Atria Health and Research Institute, New York, NY, USA

**Keywords:** Parkinson, care, model, caregiver, stigma, telemedicine, AI

## Abstract

Parkinson's disease is the fastest growing neurodegenerative disorder worldwide, yet care delivery remains fragmented and inequitable. We propose a new construct called the Parkinson's Universe. It is a person-centered model that reimagines care as a coordinated universe. This commentary summarizes the model's elements including the patient as the sun or center of the universe, the caregiver as Mercury, the social support and multidisciplinary healthcare professionals as the other planets, mission control as care coordination, stigma as Pluto, barriers as asteroids, supportive technology as satellites, and support networks as stars. We discuss clinical and policy implications, emphasizing the urgent need to move beyond the fragmented gatekeeper system to one that is proactive, equitable, and holistic. As such, the Parkinson's Universe provides a blueprint for integrating innovation, advocacy, and multidisciplinary care. The model also has relevance across other complex chronic diseases.

## Plain language summary

Parkinson's disease is the fastest growing neurodegenerative disorder, yet too many people with the condition and their families struggle to find coordinated and equitable care. We introduce the Parkinson's Universe, a new way of thinking about care that uses the metaphor of a solar system. In this model, the person with Parkinson's is the sun or center of the universe, and is surrounded by essential partners such as caregivers (Mercury), the support network and healthcare professionals (the planets), and care coordinators (mission control). Barriers to care are represented by asteroids, stigma by Pluto, and technology by satellites. The satellites are for remote monitoring or for extending support into the home. Stars symbolize support and advocacy networks that shine both light and guidance for families. By reimagining care in this way, we highlight the importance of person-centered, proactive and connected approaches. This framework not only offers hope for improving Parkinson's care, but may also apply to other complex chronic conditions.

## Introduction

**Figure 1.**
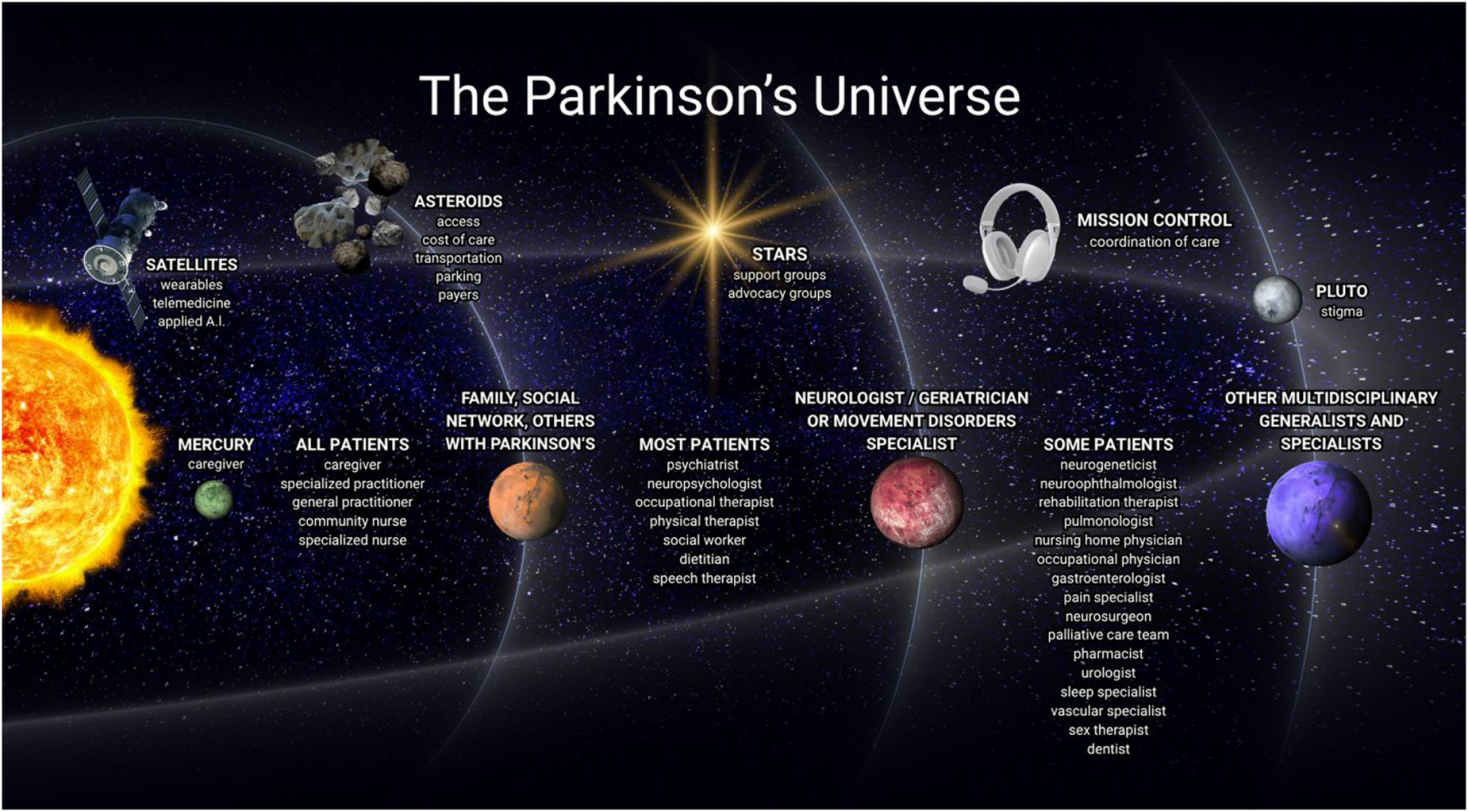
The Parkinson's Universe of Care Model. Figure provided with permission from Dorsey and Okun, The Parkinson's Plan, 2025.

Parkinson's disease (PD) is now the fastest growing neurodegenerative disorder, affecting over 11 million individuals globally.^1^ Despite advances in pharmacology and surgical interventions, care remains fragmented, poorly accessible and costly. Affected individuals and caregivers are too often left alone to navigate complex systems. We propose the Parkinson's Universe (See Figure 1), a framework for a new model that reconceptualizes care delivery as a solar system in which the person with PD is the center of the universe or the sun, and all other stakeholders, represented as planets, orbit in remarkably coordinated roles.^2^ This commentary examines the model and its implications for clinical care, policy and research.

## The core framework

### The person with PD as the sun

At the center of the Parkinson's Universe is the person as the sun.^3^ This metaphor emphasizes the centrality of the individual with PD, as the key stakeholder in this universe. Most of our efforts should be directed toward the individual with PD. Care is organized around the person's lived experience, personal goals, and core needs, in contrast to traditional gatekeeper models that prioritize providers, healthcare professionals, and insurers. Person-centered integration is both philosophically and practically essential in PD, which is a highly heterogeneous disease.^2–4^

### Mercury: the caregiver

Caregivers are represented by Mercury, the planet closest to the sun. Like Mercury, caregivers receive a lot of the heat by absorbing tremendous burdens, often silently, yet they are essential messengers and advocates.^2–4^ Mercury was the Roman god of communication, and in PD, the caregiver is an essential conduit of both connection and conveyance. We must recognize that the health hazards of being a caregiver span physical, psychological, and social domains. In some cases, being a caregiver can affect a person's long-term health as much as the disease affects the person with PD.^5^ Supporting caregivers with education, respite and financial protections will strengthen the entire PD Universe.

### The planets: family, social support, and the specialists

The closest planet to the caregiver represents family, social networks and other person's with PD. The next closest planet is a neurologist or alternatively if accessible, a movement disorders trained neurologist or a geriatrician. The final planets represent the generalists and specialists drawn from multiple healthcare disciplines. These include, among others, neurology, physical therapy, occupational therapy, speech therapy, psychiatry, and nutrition. In total, there may be over 30 different professional disciplines that can offer support for families with PD.^3,6,7^ All of these planets orbit the person with PD, as well as provide support for the caregiver. Integrated models like ParkinsonNet in the Netherlands, the Fixel Service and Science Hub Model, and the Parkinson's Foundation Centers of Excellence, all demonstrate the power of multidisciplinary care in improving outcomes and also quality of life.^2^^6–8^ These models also emphasize the added value of professional training and having deep expertise in PD management. Having access to such well-trained professionals should be a core right of every person with PD. We acknowledge that this can be challenging, especially in underserved areas of the world, so strong plans must be implemented to make the Parkinson's Universe readily and globally accessible.

### Mission control: care coordination

Who picks up the phone when a person with PD calls to say: “Houston, we have a problem”? We propose that mission control serves as the operations hub for Parkinson's care, analogous to NASA's oversight of space missions. Mission control coordinates treatment launches, sustains ongoing programs, and facilitates reintegration into society. Establishing mission control centers as standard practice could transform fragmented care into cohesive and efficient networks.^2,3,6,7^ In various parts of the world, Parkinson nurse specialists take a leading role in the functions of mission control, essentially as care coordinators.^2,6,7^ Other medical professionals can take on this role too, so long as it is clear who is responsible for this critical component of the Parkinson's Universe. A challenge for this approach is reimbursement for this coordinating role, as most medical systems only remunerate direct hands-on care delivery.

### Pluto and the asteroids: stigma and barriers

Pluto is well known in astronomy as the demoted planet. Pluto in this model represents stigma. Stigma is distant, subtle and destructive. All too often, a diagnosis of PD is incorrectly perceived by outsiders and families as the end of the world.^9^ In some cultures, a diagnosis of PD carries inappropriate negative associations such as PD being triggered by the practice of witchcraft. Shame is another critically related factor to the accumulation of stigma, and it too should be addressed.^9^ In this model Pluto, though not a planet, is always present and must be addressed as an imperative.

Asteroids symbolize barriers, and related obstructions such as limited access to care, transportation, and to the necessary finances. Asteroid fields in the Parkinson's Universe also represent some of the most dangerous obstacles: insurance systems and unsustainable costs. Addressing these obstacles will require systemic reform and advocacy.^2,8,10^

### Satellites: technology and innovation

Orbiting satellites represent innovative solutions such as telemedicine, digital monitoring using wearable sensors or smart homes, and artificial intelligence.^2^ These technologies extend care beyond the clinic and into the person's own home environment, providing real-time data as a basis for more personalized and continuous management. These technologies will be crucial for linking all elements of the Parkinson's Universe.^10,11^

### The stars: support and advocacy networks

Support and advocacy organizations are the stars of the Parkinson's Universe. Just like actual stars, there should be countless and shining initiatives which offer support for persons with disease and for affected families. Foundations and grassroots organizations can provide useful guidance, education, and empowerment. These organizations can serve to represent the voice of all those affected by PD especially with the large and powerful stakeholders, such as insurers, industries or governments. Investment in these supportive networks will ensure that no person with disease and no caregiver faces PD in isolation.^2,9,10^

## Clinical and policy implications

The Parkinson's Universe provides a philosophical shift and practical blueprint for optimal care. Implications include redefining roles to center on the needs of the individual, supporting caregivers, scaling multidisciplinary models, leveraging technology, addressing stigma and establishing mission control. Implementation of this model will require seamless collaboration across clinical, policy, and advocacy domains.^1,5^

## Challenges ahead

Despite its promise, implementation of the Parkinson's Universe model faces challenges, including workforce shortages (especially considering the rapid worldwide growth of PD cases), reimbursement structures, siloed specialties, and inequities in access. Additionally, technology adoption should raise appropriate concerns, among others, about privacy and validation. Overcoming these obstacles will demand systemic change and strong leadership.

## Conclusion

The Parkinson's Universe reframes care as a coordinated and person-centered system. By positioning the person with disease as the sun and building a supportive orbit of caregivers, specialists, mission control, technology and advocacy, this model offers a compelling vision for holistic care. If realized, this model could transform not only Parkinson's care, but also serve as a framework for other complex chronic conditions.
